# Understanding identity construction among deaf adolescents and young adults: implications for the delivery of person and family-centered care in audiological rehabilitation

**DOI:** 10.3389/fresc.2023.1228116

**Published:** 2023-11-01

**Authors:** Vera-Genevey Hlayisi, Lieketseng Victoria Sekoto

**Affiliations:** Department of Health and Rehabilitation Sciences, Division of Communication Sciences and Disorders, University of Cape Town, Cape Town, South Africa

**Keywords:** identity construction, adolescents, young adults, person and family centered care, deaf, audiological rehabilitation

## Abstract

**Introduction:**

The provision of holistic person and family-centered care in the audiological rehabilitation of adolescents and young adults (AYA) requires in-depth consideration of identity construction. The purpose of this research study was to describe the lived experience of identity construction among deaf AYA. The thoughts, perceptions, and feelings of AYA on their identity and the processes underlying the construction of identity, with a focus on navigating disability, social relations and roles, community assimilation and self-perception were explored.

**Methods:**

A qualitative interpretive phenomenological approach was adopted. Participants were a purposive sample of 5 AYA, aged 15 to 19 years. Participants had moderate to profound deafness and were enrolled in schools for the deaf where they partook in semi-structured phenomenological conversations, detailing their lived experiences with identity construction.

**Results:**

The superordinate themes of creating a self-concept, belonging, stress and being deaf emerged from participants' narratives. Identity construction occurs concurrently at several levels. At the personal level, AYA create self-conceived ideals of who they are. At relational level, identity is fostered through person-to-person and person-to-group interactions. At societal level, AYA navigate inherent challenges with hearing impairment and their positionality as deaf individuals.

**Conclusions:**

Understanding the nuances of identity construction gives insights for further research and highlights the self-ascribed identity domains and related psychosocial variables that appeal to person and family-centered care, uncovering opportunities and barriers to successful delivery. Findings have implications for the transitional care of deaf AYA that is responsive to their needs.

## 1. Introduction

The emergence of adolescence and young adulthood marks the beginning of critical developmental periods in the life course of young people. Adolescence precedes young adulthood and bridges the transition from childhood to adulthood (1, 2). Generally, the transitional nature of adolescence and young adulthood can be tumultuous. Both periods introduce innumerable biological, social, emotional, and psychological changes (2). Most importantly, adolescents and young adults (AYA) embark on identity construction, a complex and central task that requires adequate support (3). Having reduced hearing sensitivity of moderate severity or more can add to the complexity of identity construction, causing deaf AYA to interrogate ideations of who they are much earlier than their hearing peers (3, 4).

Psychologists and social scientists describe identity as an adopted self-concept influenced by the conjunction of society and self (5). Identity is also described as the outcome of individuation, a process where a unique and separate ideal of who one is to themselves, and others is created (6). Often, identity formation has long term implications on the social circles, health behaviors, career, and vocational aspirations of AYA (7). The formation of identity among AYA is acknowledged as a rite of passage to adulthood and is fostered through different processes. During identity construction, AYA assert who they are, their preferences, values, needs and how they position themselves in society (8). Consideration of these psychosocial aspects is especially important in modern-day healthcare. Discourse on honoring patient preferences, values, beliefs, and needs has taken precedence as paradigm shifts towards the biopsychosocial model of care and the conceptualization of approaches such as person and family-centered care (PFCC) have ensued over the years.

The provision of PFCC is grounded on empowering persons and families to be equal partners and collaborators in their healthcare, where their preferences, needs, beliefs and culture are upheld (9). When applied to audiological care, PFCC does not fixate on the hearing impairment or communication disorder alone but contextualizes audiological care with the broader psychosocial aspects of the person's livelihood in mind. One's identity influences their preferences, beliefs, needs and culture, thus is laden with psychosocial aspects which can inform and reorient audiological care. Notably, a critical starting point to the successful delivery of PFCC is to understand who the person receiving care is (10). However, the marginalization of the identities of deaf persons can make identity construction challenging, ultimately impeding overall quality of life (11). This among other challenges such as communication barriers, poor academic achievement, isolation, low self-esteem, reduced peer acceptance and poor mental health makes identity construction among deaf AYA challenging (12–14). An interplay between all these factors has implications for health behaviors and audiological rehabilitation outcomes.

The global need for audiological rehabilitation is on the rise. An estimated 34 million children under 15 years require audiological rehabilitation services and over 1 billion AYA are at risk for noise-induced hearing loss due to hazardous listening practices (4). Against this backdrop, AYA have been identified as an at-risk population that requires urgent and dedicated interventions for their wellbeing. Audiological rehabilitation is a continuous and holistic clinician-guided process for improving quality of life and reducing barriers to function, activity, and participation of deaf persons (15). To achieve holistic care, audiological rehabilitation interventions ought to manage the functional integrity of the auditory system, communication status and equally prioritize the psychosocial concepts that form deaf AYA's identities. Research on children with long-term health conditions and disabilities showed that proper transitional care from pediatric to adulthood care is often neglected, causing AYA who are transitioning to adulthood to regress in their rehabilitation outcomes (16). Needless to say, AYA seem to fall between the cracks, as it is not obvious the kind of care that ought to be given to this transitioning population; whose unique occupation is identity formation.

The identity formation of deaf AYA has diversified from the traditional d/Deaf perspective. Deaf is written with an upper case “D” to denote a communal identity for persons who subscribe to being part of a cultural group that uses sign language and upholds Deaf culture (17). The term “deaf” is written with lower case “d” to denote the physiological status where there is severe or complete reduced hearing sensitivity (18). To present day, deaf AYA construct a myriad of identities that can be Deaf, hearing, Hard-of-Hearing (HH), Bicultural DeaF, fluid, unresolved and dissociating from disability (10). Deaf and hearing identities are fully immersed in Deaf and hearing cultures respectively (12, 17). Hard-of-hearing AYA retain a standalone identity that also mediates and relays between Deaf and hearing worlds (13, 17, 19). Bicultural DeaF identities show Deaf pride and acknowledge and value identity formation within the hearing community (17). Fluid identities shift between identities depending on context such as whether they are aided (assuming hearing identity) or attending audiological interventions (assuming deaf identity) (13). Unresolved identities struggle with acceptance and personal adjustment, expressing confusion and grief regarding deafness, a potential indicator of identity crisis (19). Identities that dissociate from disability reject disability as an integral part of their deaf identity (12, 20).

Literature also documents the impact of school experience and cultural group dynamics on the development of identity among deaf AYA. Chen (21) indicates that whether an individual went to a Deaf, hearing, or inclusive school directly affects identity formation. One can deduce that school as a space where one predominantly develops may mold identity in the likeness of such a school space. When exploring the identity construction of HH adolescents in Canada and Sweden, researchers found that peers, educational experience and their position with the hearing and Deaf communities were influential factors (5, 19). Another factor that has implications on identity construction is the use of assistive hearing technology such as hearing aids (HAs). Hearing aid use affects domains related to identity such as communication mode, self-esteem, and physical appearance (14). Negative attitudinal perspectives of HAs may be internalized, potentially affecting personal constructions of identity and health behaviours (22). The construction of identity among AYA is an interaction of personal and external factors.

In the African and larger world context, studies generally orient around hearing status and the cultural dynamics of hearing and Deaf cultures as defining measures of identity, reinforcing a rigid view of identity. Identity formation is more complex. Evidently, identity is constructed through self-perception, engaging with disability, building relationships, adopting social roles, and assimilating into communities (17, 23–25). To support the transitional process of identity construction and meet the psychosocial needs of deaf AYA, there is a need to understand the nuances inherent in identity construction (10). Understanding identity in a more encompassing manner may help develop more personalized interventions that consider AYA in their diverse functions and capacities. Audiologists and other rehabilitation professionals require in-depth understanding of identity construction through the lived experiences of deaf AYA as this has implications for the delivery of PFCC. Exploring the lived experience not only personifies experiences but has the highest regard for the person and their lived world. On that account, this study sought to describe and understand the lived experience of identity construction among deaf AYA, using the South African context. The study described adolescents' and young adults' thoughts, perceptions, and feelings on their identity. Further, the processes underlying the construction of identity were explored, with a focus on self-perception, navigating disability, social interactions and roles and community assimilation.

## 2. Materials and methods

The research study was approved by the University of Cape Town Faculty of Health Sciences Human Research Committee, reference number: 071/2021 for meeting all ethics requirements. To invite learners from local high schools for the deaf, permission was sought and granted by the Western Cape Department of Education. Schools were auditory-verbal (oral) schools that used spoken language for learning and signing schools that use South African Sign Language (SASL). Further permission was granted by headmasters at the respective schools.

A qualitative interpretive phenomenological approach was adopted. At the core of interpretive phenomenology is a need to not only describe phenomena but interpret the meaning of phenomena according to those who experience them (26). The research study was conducted within the methodological framework of interpretive phenomenological analysis (IPA) as outlined by Smith et al. (26).

### 2.1. Inclusion and exclusion criteria

Learners who had reduced hearing sensitivity of more than 35 dB in the better hearing ear were eligible to participate (18). Records from the school audiologists were used to confirm the participants' hearing thresholds. Identity construction predominantly occurs from the ages of 10 to 25 years during adolescence and into young adulthood (27, 28). Participants who were 15–25 years could partake in the study as the study was set in high schools and would accommodate those who were older than 18 years and still enrolled. To ensure the IPA principle of sample homogeneity, participants with additional impairments such as physical, cognitive, intellectual, or other sensory impairments outside deafness were excluded from the study. Homogeneity was maintained mainly for interpretive concerns (26). Large disparities in the characteristics of the sample due to varying disabilities can diminish shared experience and shared meaning, forgoing the essence of phenomenology. Phenomenological studies may have 3–10 participants (26, 29). While the study aimed to have up to 10 participants, data saturation was attained at 5 participants, making the overall study sample size 5. It was noted that insights from the data being collected were becoming exhaustive, an indication that further enrolment and analysis were not necessary.

### 2.2. Ethical considerations

To ensure informed consent, an informative presentation was given to prospective participants in collaboration with an accredited SASL interpreter. The presentation disclosed the study aim and objectives, information about phenomenological conversations, privacy and confidentiality, the risks and benefits of the study and how collected information would be used or disseminated (30). Participants took the decision to become part of the research study on their own accord after receiving detailed information and having their queries answered. It was reiterated that participants could withdraw from partaking in the research study at any point in the study without facing any consequences.

Participants would not benefit directly from being part of the study, but research findings would lay a foundation for further research and benefit the larger community of deaf youth. Where participants experienced distress during conversations, provision for counselling was available through the school therapist. For confidentiality and privacy, all data relating to the study such as recordings, transcripts and analysis sheets were kept in a secure password protected online storage platform. The participants, as well as any identifiers were anonymized using pseudonyms in the transcripts. All study personnel (SASL interpreter and transcriber) signed binding confidentiality forms.

### 2.3. Data collection

Semi-structured, phenomenological conversations were conducted at the respective schools during the learners' free time. The school space was a comfortable and familiar setting for participants. At the schools, a secluded, quiet, well-lit, and ventilated classroom was used for these conversations. A secluded venue allowed for complete privacy for the duration of the phenomenological conversations. It was necessary to have a well-lit space to allow for optimum communication, whether signed or speech reading. A phenomenological conversation guide consisting of semi-structured open-ended questions and probe questions was used. Participants were asked to narrate as openly as possible their own personal experience with identity construction as deaf persons. Of the five participants, three were signing. Where participants were signing, the SASL interpreter relayed spoken and signed language accordingly. The duration of conversations was 45–50 min. Follow-up phenomenological conversations were scheduled when more information was needed from participants. Part of the data collection routine included writing reflections in a journal. There, I expressed where my head space was after a phenomenological conversation, what stood out and what insights I got. The reflections were also an outlet of sort; allowing me to let out any emotional or psychological baggage before moving onto the next conversations (in Supplementary Materials). Data saturation was ascertained when the explanation notes used to inform emergent themes did not yield any new information. At this point, it was noted that insights from the data being collected were becoming exhaustive and somewhat redundant.

### 2.4. Data analysis

Data analysis was completed by the researcher. To initiate the interpretation process, collected audio material was transcribed through a professional service. The audio information collected was the voice of the SASL interpreter which was simultaneously translated from SASL grammar (used by the signing participants) to spoken English. Transcripts were doublechecked for accuracy by the researcher. Field notes as well as non-verbal information such as gestures, facial expressions, moods, and emotions gathered from video recordings of phenomenological conversations were also added to give context to the phenomenological texts.

The process of interpretation used guidelines for IPA as outlined by Smith et al. (26). The initial step was reading and re-reading the individual phenomenological texts and making explanatory notes which served as initial themes. Next, emergent themes were created by clustering related or interconnected initial themes into a distinct group which best described their meaning. Lastly, abstraction involved noting interconnected emergent themes and clustering them together to form a superordinate theme (26). The abstraction process was once again repeated for the group to yield a master table of themes with initial themes, emergent themes, and superordinate themes. Analysis was concluded with member checking conversations, relayed in SASL and supported in written format. Here, the final findings and interpretations were presented to participants alongside significant transcript extracts (31). Participants were granted the opportunity to confirm, add more context or disapprove the interpretations made.

To increase confidence in the research findings, trustworthiness was applied through various strategies which were credibility, dependability, confirmability, and transferability respectively (32). For credibility, participants were given ample time to tell their narratives. Participants had the freedom to express anything they felt was necessary and follow-ups were scheduled where needed. As a means for peer reviews and reliability, debriefing meetings were held with involved senior researchers throughout the research to discuss, critique and unpack research processes and findings. To ensure dependability in the research study, being compliant with the chosen research design and all its implications was imperative (32). In terms of confirmability, a paper trail was kept of all the research processes undertaken (32). Regarding transferability, the focus of the study was not generalizability. Therefore, findings from this research study do not depict a blanket experience of all deaf AYA and should be moved into other contexts with caution.

## 3. Results

### 3.1. Participant information

Participants were a purposive sample of Deaf and Hard-of-hearing (DHH) AYA aged 15–19 years. All participants were enrolled in local schools for the deaf in Cape Town in the grades 9–11. Of the 5 participants, 3 were profoundly deaf while the other 2 were Hard-of-hearing. Table 1 below shows the demographic information of participants.

**Table 1:** Demographic information of participants in the study.

Participants (pseudonyms)	Lelethu	Juliet	Lilly	Thabo	Carren
Age (years)	17	19	16	15	18
Sex	F	F	F	M	F
School grade	11	11	9	9	11
Mode of communication	Sign language	Sign language	Spoken language	Spoken language	Sign language
Severity of deafness	Profound	Profound	Moderate-Severe	Moderate	Profound
Ethnicity	Xhosa	Biracial	Xhosa & Zulu	Xhosa	Biracial
Onset of deafness	Early	Congenital	Early	Congenital	Early
Type of deafness	Sensorineural	Sensorineural	Sensorineural	Conductive	Sensorineural
Self-ascribed deaf identity	Deaf	Deaf	Hard-of-hearing	Hard-of-hearing	Deaf
Parental hearing status	Hearing	Hearing	Hearing	Hearing	Hearing
Current assistive hearing technology	No	No	Bilateral hearing aids	Bone-anchored hearing aid	No
History of assistive hearing technology use	Yes	No	Yes	Yes	Yes
Experience with Mainstream School	No	No	Yes	No	No

**Figure 1:**
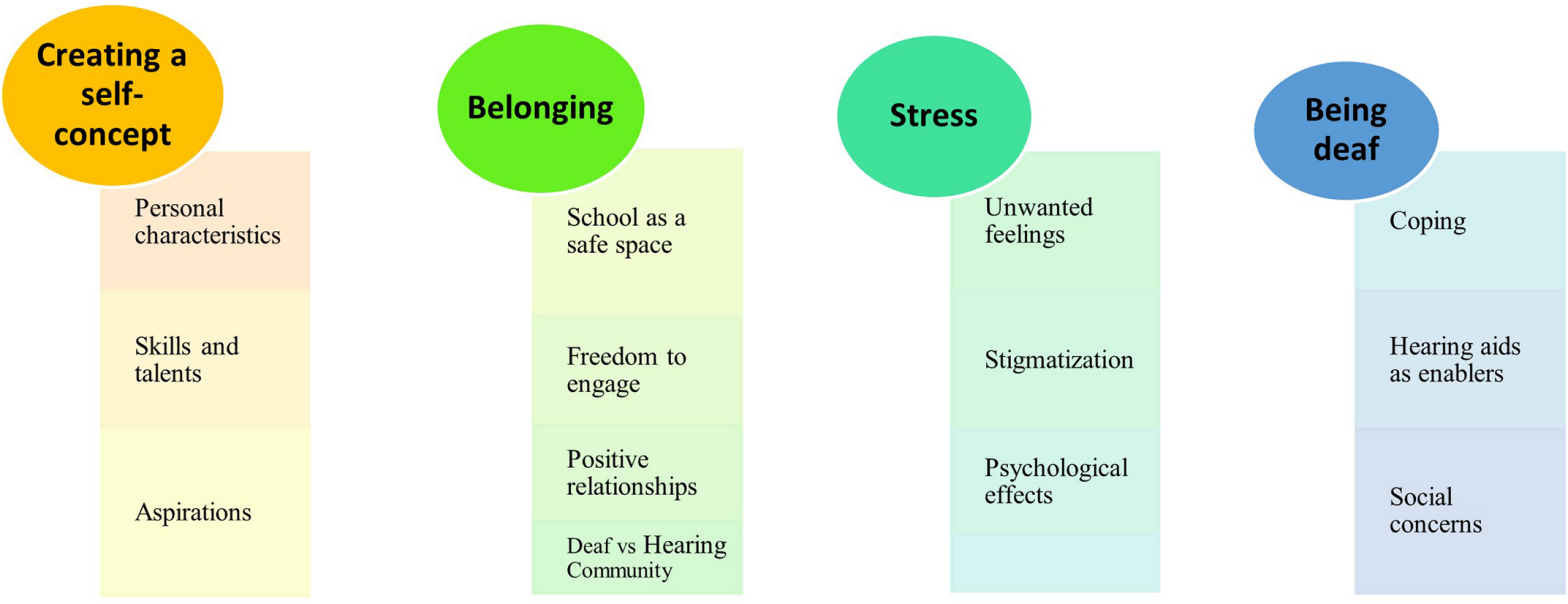
Superordinate and emergent themes.

At the conclusion of analysis, 4 superordinate themes and 13 emergent themes emerged from participants' narratives of their lived experience of identity construction. The quoted narratives of participants are written in italics. Figure 1 below shows the superordinate and emergent themes derived from the phenomenological texts of all participants.

### 3.2. Superordinate theme 1: creating a self-concept

Participants expressed their lived experience of identity construction as a process of creating a self-concept. The self-concept was an amalgamation of defining variables which pertained to the personal development of AYA's identities. The understandings were embodiments of AYA's self-identities captured in how they carried themselves (personal characteristics), the roles they assumed (skills and talents), and who they wanted to be (aspirations).

#### 3.2.1. Personal characteristics

**Thabo:** “*A person who is calm, but also strives for success.”*

**Lilly:** “*Okay. I’m a nice person. I like to play with small children and make jokes and like, go outside with children and make like lot of stuff they can make fun, and find interesting stuff to do.”*

**Juliet shared:** “*I do not really know about that, so if people want to accept me, they can and if they think I am a bad person, or if they think I am a good person. You know it is up to them to think negatively or positively. I am who I am.”*

The expression of “*I am who I am”* is meaningful. This stance was affirming of Juliet's self-ascribed identity.

Through their self-identities, participants distanced themselves from disability. Participants conceptualized their deafness as a unique trait, but not one that he limited their function.

**Juliet:** “*Deafness is a disability but for me as a person I feel like I am perfect. I can do everything except hear, it is just hearing.”*

**Thabo explained:** “*I don’t like to consider it as a disability. I just consider it as just one of those things that made me just different to other people. Yes.”*

Having a relationship with God gave participants perspective and insight about who they were. Equally, the notion of cultural identity was also regarded as representing who they are and where they come from. The spheres of identity that orient around culture, spirituality or faith were highlighted as significant aspects of identity.

**Thabo shared his perspective:** “*I think it’s important because as a person we should always have, we all … and where we come from so that’s where the factors of being Xhosa and being Christian. So, I know that I am a Christian but, and I also have an ethnic background Xhosa, so I do speak this, do speak this language and I can say that it is who I am. My background is always who I am as a person and where I come… and where I come from, so it does factor into my life.”*

**Juliet:** “*Yes I definitely have a relationship with God.”*

#### 3.2.2. Skills and talents

The skills that AYA had allowed them to push boundaries and participate in spaces that were mostly exclusive to hearing persons. Assuming leadership roles was evidence of the advanced ability to take on identities that required them to represent the interests of members of their various communities, whether at school or the larger society. Superficially, the participants were typical teenagers, enjoying lighthearted activities, but more deeply, AYA's skills and talents were reflections and expressions of their identities.

**Carren:** “*Maybe there is a medal or an award that I want to get and so I will practice here at the school and the teachers help me with that. So, I have gym and I prepare sufficiently, then they often choose me to be involved in those things with the hearing. I have competitions against the hearing people.”*

**Juliet:** “*I really love art. I grew up always loving art so now my goal is to be a designer.”*

**Lelethu:** “*I was selected as an ambassador for the Deaf people.”*

#### 3.2.3. Aspirations

There was variation and diversity in participants' prospects. The participants' self-awareness demonstrated independent thinking and engagement in the process of individuation as they had developed distinguished ideals of who they wanted to be.

**Thabo:** “*I would like to become a lawyer.”*

**Juliet:** “*Yes, I want to be an art teacher.”*

In the study, only Lilly was not yet certain of what she wanted to do in the future. This exploration phase is a necessary stage, however staying in this status for an overly extended period can be an indicator for possible identity crisis and thus could possibly become a call for concern.

**Lilly:** “*There is a lot, but I still have to choose till next year. So, I’m thinking to be a lawyer, or an airport manager, or a social worker, or a doctor. Those are the main things I’m thinking to do.”*

Through aspirations, participants wanted to be a representation. This was especially driven by feelings of underrepresentation and lack of deaf role models in the spaces they currently occupied such as school and those that they wanted to occupy in the future such as workplaces.

**Thabo:** “*I just saw that there are not a lot of people that are deaf that are lawyers, so I just want to bridge that gap a bit.”*

**Juliet: “***I am just so comfortable with you know the concept of teaching children art. As a teacher you know I would prefer that Deaf children do not have a hearing teacher, but rather that they can have a Deaf teacher. Yah, there should be more of that in schools*.”

### 3.3. Superordinate theme 2: belonging

The theme of belonging was expressed in the physical sense of being within a space that was welcoming and in the more abstract sense as seen through the actions and attitudes of others onto them. Participants acknowledged spaces, people and actions that fostered collective or communal identities. Within such settings, AYA felt seen and accepted as part of a bigger whole.

#### 3.3.1. School as a safe space

School was the predominant space within which AYA's identities were fostered and nurtured. The participants all attended special schools for the deaf, one oral and the other signing. At school AYA felt a sense of sameness, referring to the common experience of deafness. Due to longevity within the school environment, the school also became an incubator where AYA safely developed and solidified their identities along the course of time.

**Thabo:** “*You know, what I can say is that as here in this school, everybody understands what everyone is going through because we all know, we’ve all, we’ve all felt deaf, we’ve all seen, and we now know how, and everybody can just bond over that.”*

**Juliet:** “*Yah, at the school many Deaf friends. Okay so my friendship group, we have grown up together as a group. From Grade R we were very small growing up together until now you know.”*

**Lilly:** “*It’s like my home because I’ve been there since Grade R until Grade 9, so I’ve known all, most of the teachers because they were here since I was small.”*

#### 3.3.2. Freedom to engage

The freedom to engage represented participants' ability to participate without barriers in their close social circles and in the greater community. Participants also expressed how they engaged in different activities with peers or explored new connections. Exploration allowed AYA to gather the building blocks from which they constructed their identities.

When referring to how she spends time with friends **Lilly said:** *“And then we go play and do a lot of stuff man. And we help each other with work.”*

**Carren:** *“I have learnt there from so many of the different other connections that were there. They take photos of us once we are there in Nala. Oh, when I was there for the first time, you know to see all these new things, I felt so happy that was the absolute first time I have been there. As a deaf person I felt so proud to be involved in that golf experience from the school, and we won the cup.”*

#### 3.3.3. Positive relations

Good relations with friends, family and teachers were critical. They encouraged participants and helped mold them into who they wanted to be. Through these relations, they felt supported and accepted for who they are.

**Juliet: “***And my friends help me with problems, I think that they are my brothers and sisters*.”

**Juliet:** “*From school especially, yah, from school because it is the teachers who have taught me what to do and everything. They have showed me how to live as a person and what my dreams should be for the future.”*

**Lilly:** “*They always accept me the way I am. They always like call me for a conversation then I make topics and I make jokes.”*

**Thabo:** *“My relationship with my cousins is pretty good, they treat me as one of them. We are pretty close and we… they just as well, they also treat me normal. They treat me like a normal human being.”*

#### 3.3.4. Deaf vs. hearing community

Throughout the participants' narratives, Deaf and hearing communities were juxtaposed. In their perspective, there were two worlds; the deaf world, with which they were acquainted and the outside world, a reference for the hearing world to denote this existing division.

**Thabo:** “*They just call… at this school they see me as just a person who has, who is hard-of-hearing, but all the time, they don’t … but they just understand everything. But outside, people who don’t know this, they just tend to look at me—and because of this [points at hearing aid] they just tend to look at me and they just, and they wonder what is it?”*

These contrasting perspectives influenced the participants' sense of belonging. The separateness of worlds was internalized, causing participants to get the perception that in the hearing world they were in fact seen as the outsiders; denied and deprived of being a part of the outside world. There was a sense that AYA wanted to be included and recognized equally as people.

**Lelethu:** “*It is very very different the hearing and Deaf worlds. So often hearing people will tell me news or something even about drugs or something, or something or anything new information comes about. We don’t get that information, so Deaf people need encouragement.”*

**Thabo:** “*That we are deaf, but you can still be friends, we can still be people. We don’t have to be outsiders.”*

### 3.4. Superordinate theme 3: stress

The experience of identity construction was stressful for participants. As they reflected on their identity construction journeys, participants experienced many negative feelings and stigmatization. The convergence of emotional strain and stigmatization radically affected the psychological wellbeing of participants.

#### 3.4.1. Unwanted feelings

Not being able to hear and communicate left participants feeling frustrated. In these moments of frustration, participants felt very despondent, almost helpless at their situations.

**Carren:** *“So my mom, I do not actually know my mom, my mom does not actually know me, who I am as a person, so yah she struggled to teach me things.”*

**Lelethu:** *“It is very emotional and lots of ups and downs because sometimes I can’t hear.”*

**Juliet:** “*They were trying to tease me, but you know I cannot communicate with them anyway, so yah, they would laugh at me, and I would be very quiet.”*

For Lilly, the frustration of not hearing also made her shrink with embarrassment as she was faced with a teacher who scolded her for having hearing difficulties.

**Lilly:** “*So after, the teacher always shout at me that I must listen and do the work, so I didn’t, I couldn’t hear because I didn’t know that I was deaf or what’s going on.”*

#### 3.4.2. Stigmatization

In their immediate communities, participants experienced blatant discrimination and prejudice. Participants were labeled, judged and minimized by hearing peers for being deaf. The labels used to describe them had negative connotations, belittled their abilities and were said with no regard of their feelings.

**Lilly:** “*They would say that I’m dom and stupid and… I don’t know what to say.”*

**Juliet:** “*So, they would be teasing, ‘oh look! this is you sign, and you can’t hear and oh you are blind or you cannot use your voice’ and they would laugh at me.”*

**Thabo: “***There are… there were some… but not right now. There was some… so I don’t really want to explain about it*.”

Surprisingly, even when aided and capable of hearing well, Lilly was still subjected to bullying about her hearing ability. The persistence of negative beliefs even with her aided hearing abilities was evident of the deep-rooted negative attitude that exists within the hearing society.

**Lilly:** “*Because people like to say, ‘you don’t understand, you don’t hear anything!’ But I’m telling them, you won’t even hear a small sound, but I can hear it. They just don’t believe me, but I don’t know how must I explain to them.”*

#### 3.4.3. Psychological effects

The unpleasant situations that participants were confronted with left them in a state of isolation.

**Lelethu:** *“I feel isolated, there is no one there with me because before when I was younger there were people there, but now it is like everyone has forgotten about me. So, at home I just feel really alone, like never going out to visit any of my friends of enjoying myself.”*

**Lilly:** *“I was feeling so, [shaking head] not nice, because every time when I look something I couldn’t understand, or just, I can’t even talk to people or make friends or do anything with them because I couldn’t hear or do anything. So yes.”*

It was particularly hard for participants to interact in immediate spaces and in their communities which were predominantly hearing. Participants were left with no option but to withdraw from social interactions.

**Lilly:** “*In the community I didn’t like to play outside. I always sit at home and watch TV, or I just go and sleep. And they would come to me and ask ‘Are you fine or what?’ I couldn’t reply, then I just keep quiet and watch TV.”*

**Lelethu:** *“I felt that… and being adopted especially it felt that …you know… I needed an interpreter to always understand, to be able to experience life, to be able to go out and just do things. But, I cannot do that, so I’ve had to stay at home.”*

According to participants they also experienced fatigue from explaining themselves to others. This was consistently happening in their daily lives.

**Lilly:** “*Yoh, [shaking head], I would just get tired because you have to explain every time to other people.”*

**Thabo:** “*Then sometimes I get irritated always having to explain.”*

**Carren:** “*It does not really bother me. I just am kind of tired of them asking me questions, rather they ask my mother.”*

### 3.5. Superordinate theme 4: being deaf

The nature of being deaf introduced unique but shared experiences amongst participants. It meant that some experiences were inherent in their lives just by virtue of being deaf. Therefore, participants' experiences with coping, using assistive hearing devices and having social concerns were ongoing and consistently part of their being.

#### 3.5.1. Coping

Given the challenges they faced as deaf AYA, they had to find ways to adapt and cope with their challenges. Across the board, participants mostly ignored stigmatization and chose to walk away and remove themselves from prejudice-driven encounters.

**Juliet:** “*I just ignore it as that being their choice. I just continue doing what I am doing so if they tease me, it does not phase me. Yah, I go to my room or something.”*

**Carren:** “*I just ignore them, so hearing people, or yah actually I just continue walking.”*

Maintaining a positive mindset was a critical act for participants. It gave them a positive outlook on their lives, helping them to remain focused and motivated despite their hardships.

**Thabo:** “*Just tell, I will try to change that negativity, try to change it into something positive.”*

**Lilly:** *“For me, I don’t put wrong stuff like saying bad things about me—I just keep the positive stuff so I can do what I really want to do. Because if you keep saying to yourself you won’t do it and it’s just making you not going for it because nothing in the world, you just say you can’t do it, but you can do it—try your best. For yourself, not for other people.”*

#### 3.5.2. Hearing aids as enablers

Hearing aids were accepted and seen as enablers and tools for access. They granted participants opportunities and alleviated some of the limitations they experienced. Overall, participants showed a positive attitude towards HA use, showing hearing aid acceptance.

**Lilly:** “*They mean a lot because without that [shaking head] I wouldn’t be a person because that thing [referring to hearing aid] help me a lot so I can hear better and yes …”*

**Carren:** *“I became deaf from that sickness and started using a hearing aid, and then yah. So, I have been able to hear a little bit and people have taught me how to hear and to practice with the hearing aid and that has helped me.”*

#### 3.5.3. Social concerns

In their narratives, participants shared several social concerns which they felt had direct implications on them as deaf individuals. At their relatively young age, participants were already thinking about their chances of employability in the future. These thoughts emanated from knowing that employability in South Africa was potentially a challenge for them.

**Lelethu:** “*I like it because I would think there is a lot of work opportunities, there are interpreters there. So, because there is more people, there is also more access to money there, whereas in South Africa I feel Deaf people are not getting jobs but Sweden I believe it beats the Western Cape.”*

**Carren:** “*Because I have been taught so many things about my future, and I understand about my outside world and how to get access to a job. So, all those things have been really good to me.”*

Narratives reflected the dire socio-economic dynamics in their communities, which formed part of the participant's backgrounds. Where participants came from, poverty and crime were rampant. Poor socioeconomic conditions may impede access to safety and special needs that are foundational for identity construction.

**Juliet:** “*I felt there was a lot of suffering and yah it… and also hunger, not being able to have food at home. And you open the fridge and there is nothing in there and things haven’t been bought or maybe there is only soup that we keep getting all the time (teary).”*

The lack of public awareness and insight about deafness manifested into communication barriers and no accommodations for AYA. This was obviously also a major hindrance to accessing basic public services and posed a risk to their health.

**Lelethu:** “*Yes, because you need an interpreter in these sorts of instances to allow that to happen. So even as a Deaf person going to the policeman yourself, you need to use paper and write on a page to communicate with them. Even at the doctor, even if you want medication, they will give you the wrong medication and then you are sick and they don’t understand what you are saying, you have to explain so many things.”*

## 4. Discussion

An elaborate discussion of this study's findings demonstrates an in-depth understanding of the experience of identity construction for AYA who are DHH. Insights from the findings are relayed in relation to relevant scholarly literature. Further, study implications on the practice of PFCC for audiological rehabilitation as well as areas for further research are suggested.

### 4.1. Creating a self-concept

Creating a self-concept as a theme can be seen in participants elaborate reflections, self-perceptions and how they address the pertinent question on identity ‘Who am I?’ Participants in the current research study had cultivated a level of self-awareness sufficient to effectively express personal beliefs about themselves. Research shows that a self-concept comprises of personality traits, personal belief systems, physical traits, social roles and the prospective self (33). In the study, AYA defined who they were through personal characteristics, skills and talents and aspirations.

#### 4.1.1. Personal characteristics

Personal characteristics included AYA's personality traits, perception of disability, as well as culture and faith. In the current study, personality traits were attributed to AYA's temperament, their drives, likes and preferences. Historically, deficiency in the personality development of deaf children was associated with lack of spoken language, and more recently with the lack of early language acquisition and negative attitudes of caregivers towards deafness (34, 35). However, participants in the current study seemed to display positive personality development. This was evident in the way participants expressed high emotional resilience, openness to new experiences, extraversion, and agreeableness (36). For example, AYA in the current study were excited to be in spaces where they could interact with other people, were amicable, appreciated new experiences, believed in diversity.

For deaf persons, the construction of identity involves determining one's stance with the concept of disability. Disability adds dynamics to the process of identity formation, affecting one's ideations about themselves, their physicality and social groups (23). Participants in the study distanced themselves from disability as a defining component of their identities. Traditionally, the term disability has been imposed as a defining factor in the identity formation of deaf persons, often accompanied by oppressive and othering attitudes from hearing persons (37, 38). One sees how the negative perception of disability can be internalized, given the ramifications of being in a majority hearing world where the latter perspective is still prevalent. In the construction of their identity, AYA in the current study chose to accentuate two things, their normality, and abilities. Similarly, Israeli DHH young adults with in a resilience study expressed that seeing themselves as the same with everyone was good for their wellbeing (39). Furthermore, findings by Kunnen (3) and McIlroy and Storbeck (17) showed that Deaf AYA in the Netherlands and South Africa viewed themselves as fully capable people. The identity shift of AYA in the current study from disability was evident of how self-ascribed and externally ascribed identities can diverge. The act of disregarding disability as a defining component of who they are was highlighted by Murugami's (40) sentiments in his research on disability and identity, where he reiterates that identities can be constructed outside disability. Researchers in the social sciences also highlight other factors such as social roles, race, gender, religion, or ethnicity which may take precedence in identity formation (12, 25, 40, 41).

Indeed, in the current study most participants' self-concept involved a sense of identity in their religious beliefs. Equally, identification with cultural groups, being Xhosa, Zulu, mixed race or bicultural was important. Across cultures, faith and religion are enshrined in people's self-conceptualizations, mold their choices on how to navigate life and how they cope with disability (42, 43). While participants in the current study upheld their religious affiliations, they also expressed the challenge of exclusion and underexposure to religious teachings at familial or community level. This experience was similar to the experience of 15–20-year-old deaf Greek AYA who shared the same feelings in a study about religiosity (44). Feelings of indifference about religious affiliations, also came up in the current study. Minimal to no connection with religion as part of one's self-concept could be a common finding among deaf individuals. According to a Zimbabwean study, this disconnect may be due to the nature in which religious institutions have marginalized DHH persons, typically catering for the hearing population only (45).

#### 4.1.2. Skills and talents

A significant tenet of AYA's self-concepts in the current study included their skills and talents. AYA's narratives about their skills, talents and aspirations depicted AYA's present and potential social roles. To consolidate a stable self-concept, AYA must construct in abstract the person whom they will become (46). The participants in the current study framed future career roles based on their current skills. According to psychology research, the cognitive development that propels identity construction often prompts AYA to gear their actions towards attaining future goals (1). As seen in the current study, some of AYA's skills were purposefully being sharpened for utilization in the careers they would pursue. Thus, refining, and nurturing skills was critical for AYA in the current study as it translated to laying foundational blocks for the development of their future selves.

Overall, creating a self-concept, depicts the personal layer of identity that informs AYA's self-ascribed identities. The self-concept emerged highest in the hierarchy of importance among AYA's identity domains. There is a need for audiologists to be mindful of AYA's self-identity due to its direct impact on care and audiological health outcomes (47). In audiological rehabilitation, the application of PFCC is still developing with many audiologists struggling to operationalize patient centeredness (48, 49). A starting point in the delivery of PFCC among AYA can be in seeking to understand their self-ascribed identities.

Core PFCC concepts that may enhance this understanding such as consideration of the beliefs and preferences of the person being treated can seem superficial or intimidating. The *creating a self-concept* theme as a finding in the current study decodes and contextualizes *consideration of who the person is* into functional and palpable meanings for AYA who are DHH, such as their personality traits, perception of disability identity, religiosity, skills and talents and vocational aspirations. Demonstrating an awareness and understanding of AYA's self-concept is a practice of person-centeredness. When integrated into counseling strategies and other audiological interventions, an improvement in rapport, a balance in power dynamics and empowerment of AYA who are DHH may be seen. The current study findings encourage placing AYA's identity narrative at the center of their audiological care.

### 4.2. Belonging

Belonging for participants in the current study was reflected in the creation of collective identities and the push forces underlying them. A study with university young adults in America showed that a sense of belonging brought a sense of security, positive attachments and heightened meaningfulness in life (50). Likewise, experiencing a strong sense of belonging caused deaf Finnish adolescents to feel less different and just as functional as their peers (51). For AYA in the current study, the theme of belonging carried both the abstract and physical sense of feeling and being a part of a larger social circle or community. Within these communities, participants in the current study felt secure and able to foster meaningful collective and relational identities. The theme of belonging highlighted why AYA in the current study felt comfortable assimilating within their school community, friends, and family.

#### 4.2.1. School as a safe space

The school setting was an intersection for AYA's shared identities and experiences with fellow peers. All participants in the current study were enrolled in special schools for the deaf. A myriad of suitable conditions such as commonality, togetherness and being understood allowed AYA in the study to foster a sense of belonging within the school space. Olsson and Gustafsson (52) indicate that similar sentiments around commonality were shared by Swedish HH young adults who stated that they felt comfortable around other HH peers because they knew what to expect in encounters with one other. Studies that explored AYA's experiences of school life and the impact of deafness found that those who went to special schools built more friendships and felt like a part of their circles (53–56). Among the factors that impact identity construction, Chen (21) stated that the type of school attended influenced identity construction. Herein, similar observations were seen in the current study and to a positive regard.

Contradictory perspectives are presented by researchers who state that special schools deprive children from creating a sense of belonging due to limited interaction with hearing peers as well as the nature of curricula which moulds around the limitations of children with disabilities (57, 58). Consequently, some researchers in the social sciences advocate for mainstream schools, citing increased social acceptance from hearing peers and academic benefits (59, 60). Despite this, AYA with disabilities in mainstream education systems narrate feelings of isolation and poorer psychosocial wellbeing, which are potential threats for identity construction (61–63). Evidently, careful consideration is required for school placement as it could potentially compromise or enable healthy identity construction.

#### 4.2.2. Communication and exploration in identity formation

The acquisition of spoken and signed language enhanced feelings of belonging for participants in the current study. When communication efficacy was high, it made AYA feel included and truly affiliated with their communication partners. Notably communication was also a source of pride as it also represented participants' communal identities within the hearing and Deaf cultures. Additionally, the reciprocation of communication encouraged a kinship with others. For instance, the relational identity of being a friend, or a family member strengthened during these social engagements. Participants' experiences showed that being a friend and being related to accordingly as a family member shaped how they perceived themselves and where they belonged.

Communication also facilitated exploration, and the ability to explore prompted new ideas, perspectives and experiences which enriched their identity development. Through exploration with peers, participants were able to decipher what resonated with them and could be committed to vs. what to reject. Early identity theories and recent research established that for AYA to consolidate and commit to identities, exploration is a key process (8, 64). One fundamental requisite to advance exploration is the presence of a safe space (65). As shared by AYA in the current study, spaces where they could communicate well and be understood allowed them to freely evaluate personal understandings about themselves.

#### 4.2.3. Positive relations

In the current study peer relationships took precedence, followed by significant family members and educators. Positive identity development was linked to integral relationship qualities such as being supported, accepted, and being understood. Support was rendered to AYA in the current study in the form of provision for emotional, financial, and psychosocial needs. Being accepted and understood represented unconditional love and a mutual understanding that went beyond being deaf. Bain et al. (66) and Mehrad (33) state that conceptualising one's sense of belonging and identity involves a network of social relations. In a qualitative study with AYA who are DHH in the United Kingdom that explored peer relationships, participants reported supportive relationship qualities as feeling accepted and being able to relate well with peers among others (67).

Overall, the theme of belonging highlights what a conducive space for PFCC would entail, from the partners involved and the qualities that qualify individuals as important partners in the care of AYA who are DHH. These important partners form a critical part of AYA's help seeking behavior. Evidently, family and caregivers support the personal development of AYA through emotional, psychosocial, and financial support. Family-centeredness requires family and caregivers to be supported adequately by clinicians to fulfill these roles (68). However, it is important to note that the concept of family extends beyond the traditional nuclear family setting and constitutes communities and social circles where AYA feel seen, understood, and safe. The expression of the school space feeling like ‘home’ and friends being like ‘brothers and sisters’ exemplifies this.

Interestingly, peer relations take precedence to family and other caregivers. Other DHH peers can contribute insights to the care of AYA due to the value of shared experiences. Studies show that, AYA become less family-oriented in these developmental stages, becoming more peer reliant (69). Given this natural disposition to peers over family, it is critical to empower peers with the right tools to provide support to AYA. Audiological rehabilitation may incorporate peer-centric practices which may take the form of peer-focused counselling, peer-led group therapy, and peer support groups to formulate interventions that are responsive to the needs of AYA. This is a unique adjustment to PFCC that is especially necessary for this population. However, like family, the involvement of peers would also require taking the necessary precautions around confidentiality and respect for the person receiving care (70).

### 4.3. Stress

It is widely known that deafness is associated with a decline in psychosocial wellbeing (13, 14, 71, 72). Participants in the current study were subjected to general and marginalization-related stressors like stigmatization and communication barriers which caused feelings of sadness, loneliness, and frustration. Feelings of loneliness in studies with Finnish and Iranian deaf adolescents were attributed to little or no interactions with family or friends due to communication barriers and marginalizing attitudes within the hearing community (51, 73). In a study exploring everyday stressors for Israeli DHOH adolescents, participants expressed similar frustrations, reporting stress related to communication breakdown with communication partners and the negative attitudes of peers towards their deafness (74). Perpetual experience of negative emotions may not only impede positive identity construction but may leave AYA vulnerable to psychopathology in the long run.

#### 4.3.1. Stigma

Stigmatization involves the act of imposed segregation and marginalization of individuals on the basis of being deaf and stems from the perception of deafness as a liability within the hearing community (38). In the study, AYA reported being labelled, judged, and teased about their hearing disability. Substantial research investigating the effects of stigmatization on the identity construction among deaf AYA is lacking. However, collaborative research from professionals in the health and social sciences in Australia alludes to stigmatization and its impact on identity, stating that social stigma can disrupt the process of identity construction and create psychological barriers that lead to poor audiological health outcomes for DHH persons (11). According to Chapman and Dammeyer (75), stigmatization of AYA who are DHH resulted in poor health-related outcomes. Therefore, audiologists should recognize how identity issues stemming from stigmatization can disrupt the uptake of audiological rehabilitation care and be in the position to intervene accordingly.

#### 4.3.2. Psychological effects

Participants in the current study experienced isolation, and withdrawal. Participants felt that their immediate community secluded them and that they struggled to fit in. Moreover, AYA found it hard to foster and sustain peer relations within their communities due to significant communication barrier. Other researchers reported similar findings in studies where deaf adolescents were shown to experience depression, anxiety, anger, isolation, behavioral disorders, social withdrawal, and listening fatigue among others (76, 77). In the current study, participants also allude to a different form of fatigue resulting from having to explain themselves repeatedly over the span of their lives, AYA had developed a behavioral response causing reduced enthusiasm and motivation to explain themselves and their deafness. Other factors such as school placement, severity of deafness and parental communication mode were also predisposing factors for psychological issues among deaf AYA (78, 79).

Mainly, participants' stressors arise from experiencing communication barriers, a distinct challenge that can also threaten the delivery of PFCC. Participants in the current study were all born from hearing parents. Global statistics on the parental hearing status of deaf children is lacking, however, data from a study in America showed that 95% of deaf children were born to hearing parents (80). This creates a gap as AYA, and families are unable to easily foster the mutual relationships and trust on which PFCC can be founded. Beyond the functional need to communicate, AYA's narratives show that the inability to communicate has emotional and psychosocial ramifications. If unmanaged, poor communication can lead to the failure of PFCC, thus it is the duly duty of audiologists to help bridge this communication gap. In a bid to encourage active family involvement in audiological rehabilitation third party rehabilitation organizations, associations for the Deaf, social welfare programs or support groups can be utilized to capacitate families and caregivers with resources for communication, attitudinal and psychosocial barriers.

Similarly, when audiologists and other health professionals are not equipped to communicate effectively with deaf AYA, it is a major barrier that can hinder the provision of PFCC. Communication breakdown when caring for deaf persons undermines the significance of adequate communication as a patient need and identity domain. Research shows that health professionals lack deaf awareness and training in sign language, thus often experience communication difficulties with DHH persons (81). This alone diminishes the possibility for shared decision making and equal collaboration rendering the practice of PFCC virtually impossible.

### 4.4. Being deaf

What is it like for AYA to be deaf? From a phenomenological stance, deafness was not only a physiological impairment but an actual context within which participants in the study made meaning of their being. Being deaf introduced extraordinary dynamics which AYA had to learn to accept and cope with.

#### 4.4.1. Coping

In a bid to cope with stigmatization, participants tended to “*just continue walking*”. Walking away denoted low help-seeking behavior among AYA. Adolescents and young adults are said to be unlikely to ask for help regarding the challenges they experience (82). In a relatively recent study in America, most DHH young adults stated that they would not seek help despite experiencing significantly high emotional or psychological distress (83). Interestingly, deaf participants in the Crowe (83) study cited that having a strong sense of ethnic, gender, Deaf, HH or religious identity reduced the strain of their experiences, showing how positive identity construction can ease the hardships caused by stigmatization. The participants' experiences align with a study with DHH high school students in Israel. Zaidman-Zait and Dotan (74) reported that students' perception of increased stress was associated with withdrawal coping style. Although, walking away may have instantly resolved uncomfortable situations, it may not prevent future encounters or help AYA develop the capacity to handle stigmatization in a healthy manner. Participants' withdrawal coping style appeals to their assertiveness and emphasizes a need for them to learn how to manage stigma-driven occurrences in the safest and most effective way possible.

#### 4.4.2. Employability

It has been reported that deaf individuals are less likely to get jobs and retain them because of communication difficulties, limited education, lack of professional training and negative employers’ attitudes (84). Despite legislation and policies on employment equity for persons with disability, deaf persons are still severely underrepresented in job markets across the world, and of those who work, the majority occupy lower-level positions in their organizations (18, 85). The subject of employability needs further exploration. It must have been especially relevant in identity construction for it to be a common theme in AYA's accounts. Low chances of employability pose a threat to AYA's future identities.

Lack of public knowledge about deafness enabled stigmatization. Additionally, public ignorance subsequently led to lack of reasonable accommodations for deaf AYA in public spaces. According to a participant in the current study who was an ambassador for Deaf children's rights, this was a prevalent concern and major barrier to accessing public services such as hospital or police services. Social concerns were also exacerbated by the poor socio-economic dynamics in which participants in the study were brought up. Poverty in general is rife in South Africa and threatens the wellbeing of children with chronic conditions and disabilities (86). It is evident that poor socio-economic circumstances are likely to impede or limit the scope of identity construction for deaf AYA. Being deaf, the forementioned issues were all potential burdens to healthy identity construction, whose alleviation requires the implementation of tailored and evidence-based strategies.

Being deaf is a way of being that AYA cannot dissociate from. Understanding being deaf as a way of life and not just a communication disorder frames audiological rehabilitation anew, in a manner that encompasses the person in their context. It allows appreciation of the unique needs of AYA that result from their positionality and socialization in a majority hearing world. Being deaf requires AYA to cope with certain challenges, thus making positive coping strategies a need for their wellbeing. Challenges inherent in the socialization of deaf AYA such as public ignorance, lack of accommodations and poor socio-economic circumstances are not mere barriers to identity construction but threats to their livelihoods and personhoods, directly implicating PFCC.

### 4.5. Study limitations

Generalizability is not expected in phenomenological research. Participants' experiences may not reflect the experiences of all AYA who are DHH. However, findings can be contextualized to settings that are similar to the study. All participants were enrolled in special schools for the deaf. Lack of heterogeneity in the type of school attended may give a more positive perspective about the experience of identity construction due to AYA's access to early intervention, efficient communication and being in a space where they can relate with peers. Future research can enroll more participants and explore the impact of relationships, poor socio-economic background, and stigmatization on identity construction among AYA. Research evaluating peer and family-led interventions as well as programs for coping styles and assertiveness training among AYA who are DHH is necessary.

## 5. Conclusion

The experience of identity construction is layered. It is a complex and dynamic process that is actively initiated in adolescence and young adulthood. Participants' narratives gave insight into how AYA who are DHH construct their identities and further provided the nuances of why they develop certain identities. Personal ideations are most revered by AYA, amplifying their own voices against externally ascribed identities. The study objectives of understanding AYA's self-perception, social roles, relations, and community assimilation as well as navigating disability were addressed through the themes of creating a self-concept, belonging, stress and being deaf. As much as deafness poses some challenges, psychosocial variables have more significance in AYA's identity construction and overall quality of life. Understanding these psychosocial variables brings significant contribution to the practice of PFCC by contextualizing it to this unique population group. It is paramount to recognize rehabilitation professionals such as audiologists as key role players in identity construction, in their capacity to influence positive identity construction, facilitate the construction of prospective identities and mitigate identity crisis across different points of care. From a phenomenological stance, cultivating cultural humility among practitioners is prompted through reflexivity, openness and non-bias, and honoring the lived experience, thus framing anew the patient in their entirety as the person. For deaf AYA to function to their fullest capacities, audiologists should be cognizant of AYA's psychosocial needs and collaborate with other professionals and necessary stakeholders to enhance the audiological rehabilitation of AYA.

## Data Availability

The original contributions presented in the study are included in the article/**Supplementary Material**, further inquiries can be directed to the corresponding author.
