# Supplementary material for: Understanding identity construction among deaf adolescents and young adults: implications for the delivery of person and family-centered care in audiological rehabilitation
Source: Front Rehabil Sci. 2023 Nov 1;4:1228116. doi: 10.3389/fresc.2023.1228116 (PMC10646389; doi:10.3389/fresc.2023.1228116)
Supplement: Supplementary file 1 [file Table1.docx]

# **Journal entries**

*Extract from journal entry, 23/08/2021*

*I was especially nervous about this session, being my initial phenomenological conversation and long awaited. I am aware of my standing as a novel phenomenological researcher. Some things stood out for me; the sheer loneliness that Lelethu felt at home, her family situation really saddens her, and I felt it deeply. She felt that if she had not lost her parents, she would feel much better about who she was. Lelethu became very enthusiastic once she began talking about her school experiences and expressed that her friends were her support and knew who she was. She was very clear about herself as an artist and her intention to live out her artistic dreams abroad. What took me off guard was a realization of my position as a hearing individual who uses spoken English. I immediately became aware of the communication barrier that was before me in our conversation. Despite having an interpreter, the engagement I had was nothing like I imagined. This is a learning curve for me, I am aware that my approach will have to change. I will have to immerse myself in Deaf Culture and signed language grammar even in the way that I ask questions and relay information in general. These thoughts made me anxious, I wondered whether I had the will to do this. However, I am thankful to have reflected on this, it is the eye opener I truly needed.*

Extract from reflective journal entry – 25/08/2021

*Today I met Juliet. Juliet was sweet, bold and confident. Just before we started, she made a little joke and it just warmed up the entire room. What I first picked up is her passion and pride for her art skills. She has big dreams for her art career. She also has great reverence for her grandmother who was the only other Deaf person in her family; her grandmother who also introduced her to God. What really sombred today’s conversation though is learning that her grandmother had passed away. I felt the heaviness with which she talked about her passing. I sensed a deep sadness. As she narrated, her grandmother was like a parent to her and the only person to whom she could relate. I am now thinking of how loss can change the course of one’s life, how it can take away from you and leave a void that cannot be filled. I felt that the passing of Juliet’s grandmother in not so many words, had left her lonely.*

*Extract from journal entry, 15/03/2022*

*Tomorrow, I endeavor into getting feedback from participants on the study findings. I don’t know how I expected to feel; I suppose excited because it feels like such a final moment in the research study? Instead, I am such a ball of anxiety just planning and preparing for this process. Understandably so I guess, because what will participants say when they hear the exact conversations we had and how I understood them? It feels like anything could happen, and I must be ready to concede and be as graceful as possible in my reception, regardless of what it is, because the experiences of participants are bigger than me. Quite the judgment day is probably how I truly feel about this fast-approaching day, I must expect anything.*

*Extract from reflective journal entry – 06/05/2022*

*I find it very enlightening as it zooms into the core elements of what it means for deaf AYA to belong within a group; the feeling of being insiders and maybe for once, not being outnumbered, as is the case in the hearing communities where they reside. I was overwhelmed by a sense of vivid understanding which caused me to immediately reevaluate my assumptions and stance. As I was immersing myself in the participants’ narratives, I became aware of myself experiencing an insider-outsider paradox, an experience relatively similar to what participants felt as they were navigating their existence in the two words, deaf and hearing. When I placed myself within the world of AYA, I understood and embodied their perspective about the outside world, equally, I grasped the irony that comes with the outsider perceptive from the hearing world, where deaf AYA would typically be perceived as outsiders.*
